# Pain behaviors before and after treatment of oral disease in cats using video assessment: a prospective, blinded, randomized clinical trial

**DOI:** 10.1186/s12917-020-02302-w

**Published:** 2020-04-27

**Authors:** Ryota Watanabe, Diane Frank, Paulo V Steagall

**Affiliations:** Department of Clinical Sciences, Faculty of Veterinary Medicine, Université de Montréal, Saint-Hyacinthe, QC J2S 2M2 Canada

**Keywords:** Analgesia, Feline, Dentistry, Tooth extraction, Pain, Behavior, Video analysis

## Abstract

**Background:**

Specific behaviors associated with pain in cats with oral disease have not been consistently studied. The aim of this exploratory study was to identify pain-induced behaviors in cats before and after treatment of oral disease using video assessment. Twenty-four cats (6 ± 3.3 years old; 4.9 ± 1.7 kg) were included in a prospective, blinded, randomized clinical trial. Cats were equally divided into minimal (G1: minimal dental treatment) or severe (G2: multiple dental extractions) oral disease groups. After acclimation at day 0, they underwent oral examination, radiographs, scaling, and dental extractions under general anesthesia (anesthetic protocol: acepromazine, hydromorphone, propofol, isoflurane, meloxicam, and local anesthetic blocks; day 1), and were discharged at day 6. Cats were filmed remotely for 10 min using a wide-angle glass lens camera before surgery (baseline) and throughout the study at different time points (36 h of video recording). The videos consisted of four parts namely general, playing, feeding and post-feeding behaviors. A board-certified behaviorist evaluated the duration/frequency of different behaviors based on an ethogram, which were analyzed using linear mixed models and a generalized linear model, respectively (*p* < 0.05).

**Results:**

In comparison with baseline, duration of “not pawing the face” was significantly shorter at day 3 in G2. These cats spent significantly longer time “standing” and “laying” at days 3 and 6, respectively; G1 spent significantly less time “walking” and “standing” at days 3 and 4, respectively and significantly longer time “immobile” at day 3. Duration of “no/slow tail movement” was significantly longer in G2 than G1 at day 5. Duration of “pawing the ribbon” (playing) was significantly shorter in G2 than G1 at day 1. Feeding and post-feeding behaviors with soft food were not significantly different between groups or over time. Frequency of “difficulty grasping dry food” was significantly higher in G2 than G1 up to day 6. Frequency of post-feeding “head shaking” was significantly higher in both groups at day 6 when compared with baseline.

**Conclusions:**

This study identified pain-induced behaviors in cats undergoing treatment of oral disease. These behaviors may be used to differentiate painful versus pain-free cats in clinical practice.

## Background

Pain and periodontal disease affect quality of life in both human and animals [1, 2]. Periodontal disease is one of the most commonly reported diseases in companion animals [3, 4]. In cats, it produces pain, inflammation, dysphagia, halitosis, weight loss and oral hemorrhage; full-mouth extractions are commonly required as treatment [5]. However, behavioral signs of oral disease-induced pain have not been systematically investigated in cats. Current knowledge is mostly based on anecdotal evidence and review articles by experts [6–8], or studies performed in other species [9–11]. If signs of pain are not recognized, dental disease and associated pain may result in treatment delay (i.e. dental cleaning, extractions, etc.) until pain is severe, and when there is a substantial impact on the cat’s nutritional/welfare status [12]. Additionally, it is not known how behaviors associated with oral pain differ between painful and non-painful cats, and how they are affected by treatment of oral disease.

The objectives of this study were to identify the specific pain-induced behaviors associated with oral disease in cats and to evaluate the effect of oral treatment (i.e. dental extractions) on these behaviors. The hypotheses were that cats with severe disease would present specific pain behaviors that would differ in duration and frequency from cats with minimal oral disease. In addition, dental extractions would produce postoperative pain and induce the appearance of new behaviors. This exploratory study represents a follow-up report on a recent publication where pain scores and prevalence of rescue analgesia, food intake, changes in inflammatory biomarkers, and the correlation between pain and the number of tooth extractions, gingival and calculus index were studied before and after oral treatment in cats with minimal and severe disease [12].

## Results

Descriptive statistics for age, body weight, body condition score, dental score and number of extracted teeth are presented in Table 1. Cats in the minimal oral disease group were younger and lighter than those in the severe oral disease group as previously reported [12] (Table 1).

**Table 1:** Demographic data, dental score and number of extracted teeth in cats with minimal or severe oral disease

Variable	Minimal (*n* = 12)	Severe (*n* = 12)	*p* value
Gender (male, female)	Male: 3, Female: 9	Male: 9, Female: 3	
Breed	Domestic short-hair: 11 Siamese: 1	Domestic short-hair: 9 Domestic long-hair: 3	
Age (years)	3.6 (2.0)	8.5 (2.2)	< 0.0001
Body weight (kg)	4.0 (0.6)	5.8 (1.9)	0.007
Body condition score (1–9)	5 (5–6)	6 (4–6)	0.078
Dental score	1 (0–4)	17 (8–28)	< 0.0001
Number of extracted teeth	2 (0–5)	17 (8–30)	< 0.0001

Values are expressed as mean (SD) with exception of body condition score and number of extracted teeth which are reported as median (min-max)

One cat from the minimal disease group was excluded in the postoperative period due to wound dehiscence, and only preoperative data of this individual were included in the analysis. A total of 11 out of 12 cats (91.7%) in severe group received rescue analgesia on the day of dental procedure (day 1). Five videos obtained at postoperative 6 h were excluded from the statistical analysis.

The ethogram and the behaviors with low frequency (fewer than five times over a minute of observation) during video analysis that were excluded from statistical analysis are shown in Tables 2 and 3, respectively.

**Table 2:** Ethogram of general, playing, feeding and post-feeding behaviors

General	Playing	Feeding	Post-feeding
Position in the cage (D)			
Back	Pawing (D)	Eating food (D)	Grooming (D)
Front	No pawing (no interest) (D)	Not eating food (D)	Lip licking (D)
Attention to the surroundings (D)			
Looking around front of the cage	No pawing but attention to ribbon (D)	Tongue flicking (D)	Mouth pawing (D)
Not looking around front of the cage	No pawing but attention to observer (D)	Vocalization (meowing) (F)	No grooming, mouth pawing, lip licking (D)
Activity (F)			
Pawing the face	No pawing with looking away from ribbon (D)	Growling (F)	Tongue flicking (D)
Not pawing the face	Chewing ribbon (D)	Jaw quivering (F)	Teeth chattering (F)
Lip licking	Grabbing ribbon in mouth (F)	Ptyalism (F)	Jaw quivering (F)
Yawning		Difficulty grasping food (F)	Mouth opening (F)
Swallowing		Dropping food (F)	Head shaking (F)
Vocalization		Head shaking (F)	Yawning (F)
Tongue flicking		Tongue flicking (F)	Vocalization (F)
Movement (D)			
Walking		Mouth opening (F)	Swallowing (F)
Immobile		Yawning (F)	Tongue flicking (cat did not eat) (F)
Body position (D)			
Sitting		Lip licking not related to eating (F)	
Standing		Swallowing not related to eating (F)	
Laying		Vocalization not related to eating (F)	
Crouching			
Tail position (D)			
Up			
Curling around feet/body			
Tail movement (D)			
Swishing			
Not or slow movement			
Activity (D)			
Stretching			
Grooming			
Not stretching and grooming			

(D) and (F) indicate the duration and frequency, respectively.

**Table 3:** The behaviors with low frequency (fewer than five times over a minute of observation) during video analysis that were excluded from statistical analysis

General behaviors	Feeding (soft food)	Feeding (dry food)	Post-feeding (soft food)	Post-feeding (dry food)
Pawing the face	Tongue flicking	Tongue flicking	Mouth pawing	Mouth pawing
Tail up	Vocalization (meowing)	Vocalization (meowing)	Tongue flicking	Tongue flicking
Tail swishing	Growling	Growling	Teeth chattering	Teeth chattering
Stretching	Jaw quivering	Jaw quivering	Jaw quivering	Jaw quivering
	Ptyalism	Ptyalism	Mouth opening	Mouth opening
	Difficulty grasping food	Head shaking	Yawning	Yawning
	Dropping food	Tongue flicking	Swallowing	Swallowing
	Head shaking	Mouth opening	Tongue flicking	Tongue flicking
	Mouth opening	Yawning		
	Yawning	Swallowing not related to eating		
	Swallowing not related to eating	Vocalization not related to eating		
	Vocalization not related to eating			

Additional file 1 presents the *p* values for duration and frequency of some behaviors that were not statistically associated with fixed factors (i.e. group, time, group X time and gender).

Tables 4, 5 and 6 show the duration (%) of general and playing behaviors and frequency (times/minute) of behaviors in cats with minimal or severe oral disease before and after treatment, respectively.

**Table 4:** Mean (SD) of duration (%) of general behaviors in cats before and after dental treatment

Action category	Individual behavior	Time point	Minimal	Severe	*p* value between groups	*p* value compared with baseline	
						Minimal	Severe
Position in the cage	Back	Baseline	8.9 (12.1)	30.3 (36.5)	0.028^a^		
		Day 1	10.9 (30.2)	10.4 (19.9)	0.913	0.717	0.069
		Day 2	3.9 (13.0)	24.6 (40.6)	0.014^a^	0.211	0.426
		Day 3	0.0 (0.0)	16.7 (38.9)	0.031^a^	0.084	0.080
		Day 4	6.1 (20.2)	10.1 (20.9)	0.200	0.276	0.020^a^
		Day 5	1.0 (2.8)	16.7 (38.9)	0.049^a^	0.155	0.090
		Day 6	0.4 (1.4)	10.7 (17.3)	0.086	0.113	0.026^a^
	Front	Baseline	91.3 (12.1)	69.7 (36.5)	0.028^a^		
		Day 1	89.1 (30.2)	89.6 (19.9)	0.913	0.717	0.007^a^
		Day 2	96.1 (13.0)	75.5 (40.6)	0.014^a^	0.211	0.426
		Day 3	100.0 (0.0)	83.3 (38.9)	0.031^a^	0.084	0.080
		Day 4	93.9 (20.2)	89.9 (20.9)	0.200	0.276	0.020^a^
		Day 5	99.0 (2.8)	83.3 (38.9)	0.049^a^	0.155	0.090
		Day 6	99.6 (1.4)	89.3 (17.3)	0.086	0.113	0.026^a^
Attention to surroundings	Looking around front of the cage	Baseline	99.6 (1.3)	77.6 (29.4)	0.003^a^		
		Day 1	96.6 (11.4)	74.7 (40.0)	0.023^a^	0.800	0.905
		Day 2	86.5 (27.2)	69.6 (37.3)	0.021^a^	0.157	0.444
		Day 3	99.6 (1.2)	82.1 (27.8)	0.013^a^	0.948	0.602
		Day 4	94.9 (17.1)	90.6 (20.2)	0.188	0.700	0.131
		Day 5	98.9 (3.6)	83.8 (28.5)	0.034^a^	0.969	0.374
		Day 6	100.0 (0.0)	90.9 (28.7)	0.105	0.836	0.081
	Not looking around front of the cage	Baseline	0.4 (1.3)	22.5 (29.4)	0.003^a^		
		Day 1	3.4 (11.4)	25.3 (40.0)	0.023^a^	0.800	0.905
		Day 2	13.5 (27.2)	30.4 (37.3)	0.021^a^	0.157	0.444
		Day 3	0.4 (1.2)	17.9 (27.8)	0.013^a^	0.948	0.602
		Day 4	5.2 (17.1)	9.4 (20.2)	0.188	0.701	0.131
		Day 5	1.1 (3.6)	16.2 (28.5)	0.034^a^	0.969	0.374
		Day 6	0.0 (0.0)	9.1 (28.7)	0.105	0.836	0.081
Activity	Not pawing the face	Baseline	88.3 (9.7)	85.3 (14.4)	0.328		
		Day 1	85.5 (25.2)	87.5 (15.3)	0.839	0.553	0.137
		Day 2	89.6 (19.6)	74.5 (35.4)	0.017^a^	0.227	0.292
		Day 3	85.7 (28.5)	98.3 (3.0)	0.620	0.440	**0.003**
		Day 4	86.6 (17.3)	95.1 (5.5)	0.957	0.579	0.049^a^
		Day 5	82.2 (21.4)	89.8 (17.2)	0.853	0.646	0.189
		Day 6	87.6 (25.8)	85.4 (24.8)	0.342	0.382	0.367
Movement	Walking	Baseline	2.1 (1.4)	1.7 (1.8)	0.918		
		Day 1	1.3 (1.9)	4.5 (8.7)	0.266	0.056	0.846
		Day 2	1.4 (2.2)	1.4 (3.0)	0.728	0.065	0.187
		Day 3	0.4 (1.0)	0.0 (0.0)	0.889	**0.002**	0.004^a^
		Day 4	0.9 (2.5)	1.0 (1.6)	0.298	0.007^a^	0.183
		Day 5	1.5 (2.6)	0.7 (1.5)	0.863	0.101	0.075
		Day 6	1.8 (2.9)	2.7 (5.2)	0.371	0.105	0.716
	Immobile	Baseline	97.9 (1.4)	98.3 (1.8)	0.919		
		Day 1	98.7 (1.9)	95.5 (8.7)	0.266	0.056	0.846
		Day 2	98.6 (2.2)	98.6 (3.0)	0.728	0.065	0.187
		Day 3	99.6 (1.0)	100.0 (0.0)	0.888	**0.002**	0.004^a^
		Day 4	99.1 (2.5)	99.0 (1.6)	0.298	0.007^a^	0.183
		Day 5	98.5 (2.6)	99.3 (1.5)	0.863	0.101	0.075
		Day 6	98.2 (2.9)	97.3 (5.2)	0.371	0.105	0.716
Body position	Sitting	Baseline	85.0 (14.1)	58.0 (40.2)	0.037^a^		
		Day 1	63.3 (37.5)	70.8 (40.6)	0.914	0.194	0.158
		Day 2	71.3 (43.6)	58.6 (44.6)	0.166	0.516	0.811
		Day 3	83.6 (31.0)	83.3 (38.9)	0.499	0.552	0.013^a^
		Day 4	83.7 (31.5)	89.6 (20.6)	0.799	0.661	0.004^a^
		Day 5	80.0 (26.5)	76.8 (39.3)	0.396	0.091	0.075
		Day 6	87.5 (23.3)	71.8 (40.1)	0.149	0.515	0.136
	Standing	Baseline	5.6 (3.8)	3.9 (4.7)	0.640		
		Day 1	2.5 (4.5)	2.2 (3.6)	0.860	0.009^a^	0.115
		Day 2	2.1 (3.4)	3.4 (7.0)	0.480	0.008^a^	0.210
		Day 3	2.6 (6.2)	0.0 (0.0)	0.521	0.006^a^	**0.002**
		Day 4	1.4 (3.1)	1.8 (2.9)	0.357	**0.001**	0.121
		Day 5	3.7 (5.4)	2.0 (5.2)	0.792	0.054	0.081
		Day 6	3.2 (5.1)	5.8 (9.8)	0.256	0.026^a^	0.825
	Laying	Baseline	0.1 (0.2)	29.0 (38.2)	0.015^a^		
		Day 1	15.3 (34.8)	24.6 (41.5)	0.798	0.105	0.235
		Day 2	9.1 (30.2)	30.2 (45.5)	0.063	0.329	0.805
		Day 3	0.0 (0.0)	16.7 (38.9)	0.108	0.939	0.224
		Day 4	0.0 (0.0)	7.4 (18.8)	0.379	0.939	0.023^a^
		Day 5	0.0 (0.0)	8.3 (28.9)	0.352	0.939	0.028^a^
		Day 6	0.0 (0.0)	0.2 (0.8)	0.745	0.939	**0.002**
Tail position	Curling around feet/body	Baseline	33.2 (31.2)	14.5 (23.1)	0.719		
		Day 1	37.4 (46.3)	21.2 (40.1)	0.821	0.859	0.804
		Day 2	7.6 (17.7)	0.0 (0.0)	0.653	0.035^a^	0.212
		Day 3	18.2 (40.1)	25.0 (45.2)	0.140	0.190	0.428
		Day 4	18.8 (40.2)	16.1 (33.0)	0.436	0.228	0.936
		Day 5	8.1 (26.8)	20.4 (39.3)	0.052	0.029^a^	0.666
		Day 6	9.4 (29.1)	8.3 (28.9)	0.355	0.046^a^	0.569
Tail movement	No or slow movement	Baseline	54.4 (20.6)	45.0 (30.6)	0.902		
		Day 1	73.2 (40.4)	62.4 (43.5)	0.489	0.217	0.882
		Day 2	47.2 (50.5)	40.0 (48.5)	0.856	0.680	0.723
		Day 3	58.0 (47.1)	50.0 (52.2)	0.816	0.862	0.750
		Day 4	45.4 (52.1)	51.5 (50.9)	0.334	0.573	0.643
		Day 5	22.9 (35.7)	77.2 (41.7)	**0.001**	0.078	0.039^a^
		Day 6	45.3 (44.6)	49.0 (46.7)	0.515	0.655	0.844

^a^ not significant after adjustment

**Table 5:** Mean (SD) of duration (%) of playing behaviors in cats before and after dental treatment

Individual behavior	Time point	Minimal	Severe	*p* value between groups	*p* value compared with baseline	
					Minimal	Severe
Pawing ribbon	Baseline	45.0 (31.8)	15.7 (19.8)	0.018^a^		
	Day 1	46.7 (37.2)	0.4 (0.7)	**< 0.001**	0.943	0.004^a^
	Day 2	27.1 (25.7)	11.9 (17.5)	0.143	0.003^a^	0.300
	Day 3	39.4 (36.9)	14.9 (20.3)	0.054	0.208	0.739
	Day 4	33.4 (35.1)	11.4 (15.1)	0.086	0.034	0.462
	Day 5	36.7 (36.6)	12.4 (18.1)	0.059	0.087	0.474
	Day 6	33.4 (29.5)	5.9 (15.2)	0.013^a^	0.064	0.025^a^
No pawing but attention to ribbon	Baseline	13.6 (15.9)	22.3 (12.5)	0.031^a^		
	Day 1	10.0 (15.9)	15.3 (20.1)	0.0001^a^	0.610	0.002^a^
	Day 2	10.0 (18.1)	14.6 (16.9)	0.016^a^	0.718	0.748
	Day 3	5.6 (10.2)	15.4 (15.8)	0.016^a^	0.672	0.808
	Day 4	3.9 (5.9)	22.4 (24.3)	0.026^a^	0.506	0.665
	Day 5	4.8 (8.1)	16.9 (19.5)	0.024^a^	0.405	0.580
	Day 6	2.7 (4.1)	19.9 (23.8)	0.010^a^	0.427	0.824

^a^ not significant after adjustment

**Table 6:** Mean (SD) of frequency (times/min) of behaviors in cats before and after dental treatment

	Individual behavior	Time point	Minimal	Severe	*p* value between groups	*p* value compared with baseline	
						Minimal	Severe
Feeding (dry)	Difficulty of grasping food	Baseline	0.3 (0.6)	1.3 (1.8)	**0.005**		
		Day 6	0.2 (0.7)	2.0 (2.1)	**0.001**	0.376	0.156
Post-feeding (dry)	Head shaking	Baseline	0.1 (0.3)	0.2 (0.4)	0.622		
		Day 6	0.5 (0.7)	0.7 (1.1)	0.733	**0.001**	**0.005**

### General behavior

In comparison with baseline, duration of “not pawing the face” was shorter at day 3, and “standing” and “laying” were longer at days 3 and 6, respectively in the severe group; duration of “walking” was shorter at day 3, “immobile” was longer at day 3 and “standing” was shorter at day 4 in the minimal group (Table 4). Duration of “no/slow tail movement” was longer in the severe than in the minimal group at day 5 (Table 4). The expected occurrence of duration of “tail curl” was significantly higher in female than male (*p* = 0.017).

### Playing behavior

Duration of “pawing the ribbon” was significantly shorter in the severe group than in the minimal group at day 1 (Table 5).

### Feeding behavior

#### Dry food

Cats in the severe group had significantly higher frequency of “difficulty grasping dry food” than in the minimal group up to day 6 (Table 6). This specific behavior was observed more commonly in males than females (*p* = 0.029).

### Post-feeding behavior

#### Dry food

Frequency of post-feeding “head shaking” was significantly higher in both groups at day 6 when compared with baseline (Table 6).

Additional file 2 includes a video with a summary of behavior changes and results of the study in cats with minimal or severe oral disease.


**Additional file 2:** Summary of behavior changes and results of the study in cats with minimal or severe oral disease. A 10-min video consisted of 4 parts including general, playing, feeding and post-feeding behaviors. Cats with severe oral disease were less active, less playful and had more difficulty grasping dry food.


## Discussion

This study identified specific pain-induced behaviors associated with oral disease in cats undergoing dental treatment. According to our hypotheses, these behaviors differed between cats with minimal and severe oral disease, and new behaviors appeared after the dental procedure due to postoperative pain [12]. Overall, cats with severe oral disease were less active when compared with baseline or cats with minimal oral disease. For example, duration of “walking” and “standing” was shorter whereas they were more reluctant to move (“immobile” and “no/slow tail movement”) than in the minimal group at specific time points postoperatively. Additionally, postoperative pain induced changes in grooming. Duration of “not pawing the face” was shorter in cats with severe oral disease after the dental procedure than baseline. Less activity was also observed with these cats: duration of “standing” and “laying” was longer after dental extractions than before the procedure.

Some studies have evaluated oral pain when comparing the efficacy of different analgesic treatments in dogs and cats [13, 14]. In the current study, the CMPS-F was used for pain assessment. This tool has been widely used for feline acute pain evaluation, and theoretically, it can be applied for different sources of pain [15]. The UNESP-Botucatu multidimensional composite pain scale for feline pain assessment [16] has only been validated in cats undergoing ovariohysterectomy and the authors opted to use the CMPS-F in this study. However, the authors found some limitations when using the CMPS-F to evaluate oral pain in this study. None of the cats scored points for questions 3 (ignoring any wound or painful area: 0 points or attention to wound: 1 point) or 6 (after gentle pressure of the wound, does the cat?: do nothing – 0 points; swish tail/flatten ears – 1 point; cry/hiss – 2 points; growl – 3 points; bite/lash out – 4 points). Therefore, it may be difficult to predict how cats would give attention to wound for question 3 in dental pain. Indeed, an opposite finding would be expected when cats are painful. Additionally, most cats do not appreciate palpation of the mouth area before or after the dental procedure for question 6. An escape behavior was often noticed but none of the behaviors of CMPS-F question 6 was easily detected. Based on this rationale, it is possible that pain was underestimated in some cats when they were less active and reluctant to move. The pain-induced behaviors reported here may add additional information to feline pain assessment in dentistry and clinical practice.

The study presented an ethogram of normal and those behaviors that are presumed to be affected by oral disease based on previous reports and clinical experience [6, 7, 17]. However, some of these behaviors are also known to be influenced by the cats’ demeanor [18]. For this reason, cats with shy or fearful behavior were excluded to minimize bias and overestimation of pain scores during assessment.

The duration of “pawing the ribbon” was significantly shorter in the severe group than in the minimal group. Additionally, albeit not significantly, the duration of “no pawing but attention to ribbon” was always longer in severe than in the minimal group. These playing behaviors were affected by oral pain after dental treatment; painful cats with severe oral disease were less playful. On the other hand, playing is a unique feature of each cats’ demeanor and temperament, which could be affected by pain, but also stress, anxiety and hospitalization. This may be the reason why the duration of other playing behaviors was not always significantly different between groups or baseline values (i.e. “chewing the ribbon” and “grabbing in the mouth”). Therefore, changes in playing behavior may be more important in the home environment than in the hospital setting.

Cats with severe oral disease showed significant differences in feeding behavior when compared with cats with minimal disease. These differences were also observed in both groups for “head shaking” during post-feeding behavior assessment on day 6. The behavior “head shaking” was probably evoked by pain during feeding since severe acute inflammation is present in the first postoperative days. Chewing the dry food by using the remained teeth but also the gingiva/wound where teeth were extracted may produce pain. Our previous study showed that the amount of dry and soft food intake for 3 min, and dry food intake for 2 h were significantly decreased in cats with severe oral disease [12]. The study concluded that cats with oral pain require longer periods of time to eat both dry and soft food than those with minimal pain. Frequency of “difficulty grasping dry food” was observed more commonly in males than females. This could be explained by the unequal distribution of male and female cats in the study (3 males and 9 females in minimal group and 9 males and 3 females in severe group). Therefore, this result may show that cats with severe disease had more “difficulty grasping dry food” than those with minimal disease, and may not have a direct association with sex per se.

This study has some limitations: 1) palpation of the painful area (question 6 of CMPS-F) was performed over the lips since direct palpation of gingiva would not always possible due to some cat’s temperament. Additionally, this would have unmasked the observer to the dental severity group; 2) many behaviors were not significantly different between groups for duration and frequency. In this case, the number of behaviors analyzed using the ethogram and the rigorous statistical approach with many group comparisons followed by sequential adjustment resulted in a decrease of the significant “real” *p* value. It seems that this is not a specific issue to our study or in cats, and it could be also related to duration of filming. For example, previous studies could not find significant differences in the frequencies of specific behaviors in rats or bears with oral pain when duration of filming was short (7 and 15 min, respectively) or similar to our study (10 min) [10, 11]. On the other hand, frequencies of oral pain behaviors were found in ferrets when using longer filming periods (1 h for each time point) than the present study [9]. Therefore, duration and frequency of other specific behaviors could exist in cats with oral disease if duration of filming was longer than in this study. 3) 11 out of 12 cats in severe group received rescue analgesia at day 1 when postoperative acute pain and inflammation is severe. Five of these videos of painful cats were excluded from the analysis after the administration of hydromorphone since this could have biased video assessment [19]. This high prevalence of rescue analgesia in the severe group on day 1 may have underestimated our video observations. In other words, some differences could have been detected between disease severity groups, and day 1 in comparison with baseline if these videos had not been excluded. 4) there were several behaviors in the study that were no longer significant after statistical adjustment due to the numbers of comparisons. This could have led to a type II error where a difference between disease severity groups existed, but this hypothesis was rejected after sequential adjustment. Perhaps, this may be the main reason why some of the behaviors were not statistically significant even when they could be of clinical relevance. This included “position in the cage” (i.e. duration in the “back of the cage”), “attention to surroundings” (i.e. duration of “not looking around front of the cage”), and “body position” (i.e. “laying”), and playing behaviors (i.e. “pawing ribbon” and “no pawing with attention to ribbon”).

## Conclusion

This study identified some pain-induced behaviors in cats undergoing treatment of oral disease that can be used to differentiate painful versus pain-free cats, and as indicators of acute pain in these patients. Overall, cats with severe oral disease were less active, less playful and had more difficulty grasping dry food.

## Methods

### Study design

This study was approved by the Institutional Animal Care and Use Committee of the Université de Montréal (protocol 17-Rech-1890) and performed at the Centre hospitalier universitaire vétérinaire (CHUV), Faculty of Veterinary Medicine, Université de Montréal, between July 2017 and February 2018. This clinical trial is reported in accordance with the CONSORT guidelines [20]. The study design was a prospective, blinded, randomized clinical trial.

### Animals

**Fig. 1:**
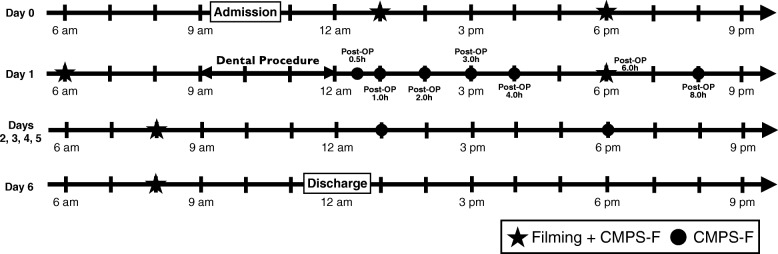
Example of a timeline for pain assessment and video filming in cats undergoing oral treatment for 7 days. CMPS-F: Glasgow composite measure pain scale-feline

Twenty-four adult (> 1 year of age) cats of any breeds and gender with or without naturally occurring oral disease were included. Cats that could possibly require oral treatment were recruited from different shelter facilities. Before enrollment, an oral examination including the condition of gingiva and the amount of calculus was performed in the conscious cat by the dentistry service so the principal investigator (PS), but not other observers involved with anesthesia and pain assessment, would have an idea of group allocation that could facilitate further patient recruitment (cats with minimal or severe disease). A written informed consent was obtained before enrolment in the study. Animals were admitted approximately 24 h before general anesthesia (day 0); dental treatment was performed on day 1. Cats were discharged on day 6 (7 days after arrival and 6 days after treatment of oral disease) (Fig. 1). During hospitalization, they were housed in stainless steel cages in the cat ward of the CHUV with access to water ad libitum, toys, litter box and bedding. At the end of the study, they were returned to the shelter facilities for adoption.

### Inclusion and exclusion criteria

Cats with body condition score ranging from 4 to 6 out of 9 and with no/minimal or severe oral disease that would require oral treatment including dental examination, scaling and/or extractions were included in the study. Inclusion criteria were also based on history, medical records, complete physical examination, and hematology and biochemical panel. Cats presenting fearful behaviors, concurrent medical conditions, systemic disorders (e.g. cancer, renal, cardiovascular, hepatic, or gastrointestinal disease) were not included. Cats were excluded if they received any medication including analgesics and antibiotics for up to 10 days before the study had begun or presented signs of disease during hospitalization.

### Treatment of oral disease

#### Group allocation

Complete dental examination and radiography were performed, and patients underwent dental scaling and dental extractions (if needed) by a board-certified dentist and a resident of the American Veterinary Dental College. Group allocation (i.e. minimal or severe oral disease) was determined according to a scoring system suggested by these two individuals in agreement with the principal investigator (PS) based on their previous clinical experience. In brief, the number and location of extractions were thought to be important in determining the possible severity of postoperative pain [12]. The scores were as follows: canine tooth - 3 points, third premolar of maxilla or molar of mandible - 2 points, second premolar of maxilla or premolar of mandible - 1 point; a score of 2 points was given if seven or more incisive teeth and/or first premolars of the mandible were extracted; otherwise a score of 1 point was given if six or fewer incisive teeth were removed. The total dental score was calculated, and cats were allocated to the minimal oral disease group if dental score ≤ 7, and to the severe oral disease group if dental score was ≥8.

#### Anesthesia and analgesic protocol

Premedication consisted of intramuscular (IM) (i.e. epaxial muscles) administration of acepromazine (0.02 mg/kg; 1 mg/mL, Acepromazine maleate, Gentès & Bolduc, Saint-Hyacinthe, QC, Canada) and hydromorphone (0.1 mg/kg; 2 mg/mL, Hydromorphone hydrochloride, Sandoz, Boucherville, QC, Canada). A eutectic mixture of local anesthetic cream (EMLA cream lidocaine 2.5% and procaine 2.5% cream, Astra Zeneca, Mississauga, ON, Canada) was applied to the skin over the cephalic vein after clipping and covered with plastic film and adhesive bandage. Approximately 20 min later, a 22-G intravenous (IV) catheter was aseptically placed in one of the cephalic veins. Anesthetic induction was performed with the administration of intravenous propofol (4.0 ± 1.2 mg/kg) (10 mg/mL, Propoflo 28, Zoetis, Kirkland, QC, Canada) until the anesthetic depth for ﻿endotracheal intubation was achieved . The arytenoid cartilages were splashed with 0.05 mL of lidocaine 2% (Lidocaine hydrochloride sterile injection, 20 mg/mL, Vétoquinol N.-A.Inc., Lavaltrie, QC, Canada), and cats were intubated with a cuffed endotracheal tube and connected to a coaxial Mapleson D system. Anesthetic maintenance was performed with isoflurane (Isoflurane USP, Fresenius Kabi, Toronto, ON, Canada) in 100% oxygen. Anesthetic monitoring was performed with a multiparametric monitor (Lifewindow 6000 V Veterinary Multiparameter Monitor; Digicare Animal Health, Boynton Beach, FL, USA) as reported in our previous article [12]. A crystalloid solution was administered (2–5 ml/ kg/hour) throughout the procedure. Cats received local anesthetic blocks with bupivacaine 0.5% (50 mg/mL, Sensorcaine, AstraZeneca, ON, Canada) using a 25-G needle if dental extractions were required. These included the mental, infraorbital, maxillary and/or inferior alveolar mandibular nerve blocks approximately 20 min before tooth extraction. The total dose of bupivacaine for all anesthetic blocks did not exceed 2 mg/kg. Meloxicam (0.2 mg/kg; Metacam 5 mg/mL Solution for Injection; Boehringer Ingelheim, Burlington, ON, Canada) was administered subcutaneously at the end of the dental procedure. Three additional doses of meloxicam at 0.05 mg/kg were administered orally at 24, 48 and 72 h after the first dose according to label recommendations in Canada.

#### Video recording

The schedule for video recording was performed according to Fig. 1. There were nine time points of video recording and each lasted 10 min (total of 90 min for each cat). A wide-angle glass lens camera (GoPro Hero 5, GoPro, Riverside, CA, USA) set between cage bars was used. Cats were moved to a specific cage for video recording that included better lighting and material quality. After a 5-min acclimation period, the camera was activated remotely using a smart-phone (iPhone7, Apple Inc., Cupertino, CA, USA). During the 10-min period, video recording was performed as follows: a) time 0–3 min: the general behaviors of the cat were recorded without any observer in the room (3 min; general behavior), b) time 3–5 min: the observer entered the room, greeted and petted the cat, stimulated the cat to play with a ribbon toy (2 min; playing behavior), c) time 5–8 min: the cat was fed with dry or soft food; feeding should potentially evoke pain behaviors as it has been described in the literature (3 min; feeding behavior) [6] and d) time 8–10 min: food was removed, and cats were filmed for another 2 min without the observer in the room (2 min; post-feeding behavior). Cats were fed with dry food (Hill’s Science Diet, Adult Optimal Care – Dry; Hill’s Pet Nutrition Canada Inc., Mississauga, ON, Canada) at 6 pm on day 0 and 8 am on day 6. A commercial canned prescription recovery diet (Hill’s Prescription Diet a/d; Hill’s Pet Nutrition Canada Inc., Mississauga, ON, Canada) was provided at 1 pm on day 0; 6 am and postoperative 2 and 6 h on day 1; at 8 am, 1 pm and 6 pm at days 2, 3, 4 and 5. Any remaining food was removed after 2 h.

#### Video analysis

A total of 36 h of video material was analyzed using a professional software (The Observer XT, Noldus information technology, VA, U.S.A). Videos were randomized according to the website www.randomization.com and assessed by a board-certified behaviorist [DF] of the American College of Veterinary Behaviorists who was blinded to severity groups. An ethogram was developed using normal behaviors and those described in painful cats with oral disease in review and scientific articles, textbooks and clinical experience [6, 7, 17]. Some behaviors were added to the ethogram based on the researchers’ observation during pain assessment of these cats [12]. The duration (%) (duration of each behavior/video length × 100) or frequency (times of the event/minute or total number of each behavior during the video/video length) for each behavior were obtained for statistical analysis. Baseline duration and frequency of each behavior were calculated using the mean of preoperative values. For general and playing behaviors, the mean of three values were used (1 pm and 6 pm on day 0 and 6 am on day 1) whereas for feeding and post-feeding with soft food, the mean of two values (1 pm on day 0 and 6 am on day 1) were used to calculate baseline mean values. The behaviors that were recorded less than five times during video assessment were excluded from statistical analysis.

#### Pain assessment

Pain assessment was performed by an observer [RW] who ﻿was unaware of the disease severity using the Glasgow composite measure pain scale-feline (CMPS-F) according to Fig. 1 [15]. Pain assessment was performed before video recording. Rescue analgesia was administered with hydromorphone at 0.05 mg/kg IV (if the intravenous catheter was in place, first 24 h after surgery) or 0.1 mg/kg IM (if the intravenous catheter had been removed) when CMPS-F scores were ≥ 5/20 at any time during the study. Based on the duration of hydromorphone in cats, the videos obtained within 2 h of rescue analgesia were excluded from statistical analysis to avoid bias [19].

### Statistical analysis

Statistical analyses were performed using SAS version 9.3 (SAS Institute, Cary, NC, USA). The power analysis revealed that eight cats would be needed per group to detect a difference of three points in the CMPS-F pain scores between the two groups 80% of the time using an alpha value of 0.05, and a standard deviation within group of two points [12]. Twelve cats were included per group for adequate power considering the individual variability of oral disease. After normality test using a Shapiro-Wilk test, demographic data for each treatment group were compared using two-sample t-tests or Mann-Whitney U where appropriate. Duration and frequency of each behavior were compared between groups at each time point, and between baseline and the postoperative time points in both groups. Duration of each behavior was transformed using the arcsine square root transformation and analyzed using a linear mixed model with patient identification as the random factor, and groups and time and their interaction as fixed factors, and gender as co-factor. Frequency of each behavior was analyzed using a generalized linear model with log link and Poisson errors with patient identification as the random factor, groups and time as fixed factors, and gender as co-factor. When there was an association with fixed factors, a series of a priori contrasts were performed to compare the means using sequential Benjamini-Hochberg’s adjustment. *p* < 0.05 was considered statistically significant.

## Supplementary information

**Additional file 1: ***p* values of behaviors that were not significantly associated with fixed factors. Table with individual behaviors and p values for group, time, group x time and gender comparisons.

## Data Availability

The datasets used and/or analyzed during the current study are available from the corresponding author on reasonable request.

## Abbreviations

CHUV: Centre hospitalier universitaire vétérinaire
CMPS-F: Glasgow composite measure pain scale-feline
IM: Intramuscular
IV: Intravenous
