# Supplementary material for: Pain behaviors before and after treatment of oral disease in cats using video assessment: a prospective, blinded, randomized clinical trial
Source: BMC Vet Res. 2020 Apr 27;16:100. doi: 10.1186/s12917-020-02302-w (PMC7146962; doi:10.1186/s12917-020-02302-w)
Supplement: Supplementary file 2 — Additional file 1: p values of behaviors that were not significantly associated with fixed factors. Table with individual behaviors and p values for group, time, group x time and gender comparisons. [file 12917_2020_2302_MOESM1_ESM.docx]

Additional file 1: *p* values of behaviors that were not significantly associated with fixed factors.

| **­** | **Individual behavior** | **Group** | **Time** | **Group × time** | **Gender** |
| --- | --- | --- | --- | --- | --- |
| General | Lip licking | 0.164 | 0.352 | 0.256 | 0.745 |
|  | Yawning | 0.295 | 0.695 | * | 0.767 |
|  | Swallowing | 0.217 | 0.523 | 0.790 | 0.838 |
|  | Vocalization | 0.277 | 0.065 | * | 0.988 |
|  | Tongue flicking | 0.329 | 0.194 | 0.567 | 0.514 |
|  | Crouching | 0.973 | 0.914 | 0.186 | 0.407 |
|  | Grooming | 0.293 | 0.331 | 0.780 | 0.156 |
|  | Not stretching and grooming | 0.296 | 0.351 | 0.794 | 0.149 |
| Playing | No pawing (no interest) | 0.187 | 0.081 | 0.667 | 0.183 |
|  | No pawing but attention to observer | 0.580 | 0.071 | 0.451 | 0.901 |
|  | No pawing with looking away from ribbon | 0.756 | 0.929 | 0.385 | 0.996 |
|  | Chewing ribbon | 0.065 | 0.163 | 0.185 | 0.566 |
|  | Grabbing ribbon in mouth | 0.060 | 0.092 | * | 0.857 |
| Feeding (soft food) | Eating food | 0.585 | 0.170 | 0.566 | 0.362 |
|  | Not eating food | 0.602 | 0.222 | 0.438 | 0.571 |
|  | Tongue flicking | 0.265 | 0.485 | * | 0.204 |
|  | Lip licking not related to eating | 0.928 | 0.150 | * | 0.210 |
| Feeding (dry food) | Eating food | 0.072 | 0.430 | 0.731 | 0.831 |
|  | Not eating food | 0.137 | 0.318 | 0.838 | 0.799 |
|  | Dropping food | 0.736 | 0.313 | * | **0.029** |
|  | Lip licking not related to eating | 0.314 | 0.181 | * | 0.364 |
| Post-feeding (soft) | Grooming | 0.567 | 0.317 | 0.105 | 0.987 |
|  | Lip licking | 0.857 | 0.726 | 0.929 | 0.646 |
|  | No grooming, mouth pawing, lip licking | 0.946 | 0.736 | 0.329 | 0.506 |
|  | Head shaking | 0.745 | 0.933 | 0.070 | 0.411 |
|  | Vocalization | 0.165 | 0.292 | 0.374 | 0.340 |
| Post-feeding (dry) | Grooming | 0.700 | 0.055 | 0.374 | 0.947 |
|  | Lip licking | 0.802 | 0.612 | 0.985 | 0.341 |
|  | No grooming, mouth pawing, lip licking | 0.978 | 0.622 | 0.410 | 0.243 |
|  | Vocalization | 0.411 | 0.706 | * | 0.385 |

* The model failed to converge.
